# Application of Self‐Expandable Metal Stent in Dogs and Cats for the Management of Extrahepatic Biliary Obstruction: 13 Cases (2021–2024)

**DOI:** 10.1002/vms3.70548

**Published:** 2025-08-09

**Authors:** Suh Hyun Chai, Kyuseok Choi, Sungin Lee, Yongsun Kim

**Affiliations:** ^1^ Department of Small Animal Surgery BON Animal Medical Center Suwon Republic of Korea; ^2^ Department of Veterinary Surgery, College of Veterinary Medicine Chungbuk National University Cheongju Republic of Korea

**Keywords:** cat, dog, EHBO, extrahepatic biliary obstruction, self‐expandable metal stent

## Abstract

**Objectives:**

To describe the clinical characteristics of 11 dogs and 2 cats with extrahepatic biliary obstruction (EHBO) who underwent self‐expandable metal stent (SEMS) placement. We also examined the indications for the procedure and evaluated its outcomes. Lastly, we identified risk factors associated with survival in these animals.

**Methods:**

This retrospective study included 11 client‐owned dogs and 2 client‐owned cats that underwent biliary SEMS placement. Medical records were reviewed.

**Results:**

The average age of the animals was 9.3 years (range, 6–15). The majority of animals had at least one clinical sign associated with EHBO. Frequently appearing clinical signs were inappetence, lethargy and jaundice. No complications were found related to the stenting procedure. Clinical signs and serum total bilirubin concentration improved postoperatively in all 13 animals. The 12 animals (92.3%) that survived until discharge were followed up to 2 years postoperatively, during which time no significant complications were observed.

**Clinical significance:**

Biliary SEMS placement may be considered as a treatment option for dogs and cats with EHBO, with minor complications and potential for permanent treatment of EHBO.

## Graphical abstract

Extrahepatic biliary obstruction in dogs and cats is life‐threatening. Biliary self‐expandable metal stents (SEMS) provide a minimally invasive treatment, effectively relieving the obstruction with fewer complications. SEMS offers the potential for long‐term or permanent relief, significantly improving outcomes and quality of life in affected animals.

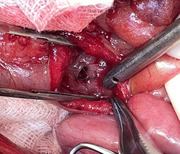

## 1 Introduction

Biliary intervention using self‐expandable metal stents (SEMS) is the modality of choice for relieving malignant obstructions within the common bile duct (CBD) in humans (Nam and Kang 2016). This minimally invasive procedure not only offers a better alternative to open surgeries, proven with overall reduced patient morbidity and mortality, but also has several advantages over conventional red rubber and polyethylene stents, including higher radial force, longer patency, lower occlusion and migration rates, and a decreased need for reintervention (Lee et al. 2016). However, to date, only limited information is available on biliary SEMS placement in animals with extrahepatic biliary obstruction (EHBO).

EHBO is a life‐threatening condition in dogs and cats in which bile flow is obstructed along its passage from the liver into the duodenum, via the CBD and major duodenal papilla (MDP) (Mayhew et al. 2006). Owing to the complex physiology and anatomy of the hepatobiliary system, EHBO may develop from both malignant and benign causes, with pancreatitis being the most common, followed by cholelithiasis, cholangitis, cholecystitis, inspissated bile, biliary ductal sludge or mucocele and neoplasia (Mayhew et al. 2006).

**FIGURE 1:**
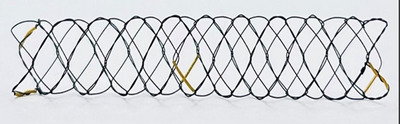
Metallic biliary stent (S&G Biotech, Seoul, Korea) sized 6 mm (diameter) × 20 mm (length), with 4 mm‐wide cells, single‐bare, shape memory nitinol alloy.

Biliary stents play a key role in rapidly restoring bile flow in dogs and cats with EHBO that is unresponsive to medical treatment by providing mechanical bypass to the obstruction (Griffin et al. 2021). Therefore, stent selection must be precise based on the degree of dilatation, location and length of the obstruction within the CBD (Lee et al. 2016). To date, data only on the use of red rubbers or polyethylene pigtail catheters in dogs and cats with EHBO have been published (Boulay et al. 2010). In the current study, both a commercially available human SEMS and a newly designed animal‐specific SEMS (Figure 1; single bare metallic biliary stent, S&G Biotech, Seoul, Korea) were applied to dogs and cats diagnosed with EHBO requiring mechanical biliary bypass.

The present retrospective study aimed to describe the clinical characteristics and outcomes of the dogs and cats who received SEMS for the treatment of EHBO and to evaluate potential risk factors for postoperative survival in these animals.

## 2 Materials and Methods

### 2.1 Case Selection and Data Collection

The electronic medical records of all animals that were treated with biliary SEMS as part of EHBO management at a veterinary medical centre between March 2021 and December 2024 were reviewed. SEMS was selected if there was a stricture, mass or biliary ductal debris, which had potential for recurrence of EHBO.

All cases of SEMS placement were client‐consented prior to surgery. Cases were included if the animals underwent exploratory laparotomy prior to biliary stenting and if a complete medical record was available for review. Records were excluded from the study if the selection of the stent was deemed inappropriate for the specific animal or if the medical record was incomplete.

Medical records included information on signalment, history of hepatobiliary diseases, clinical signs, findings on physical examination, laboratory tests, diagnostic imaging and postoperative outcomes.

### 2.2 Procedure

**FIGURE 2:**
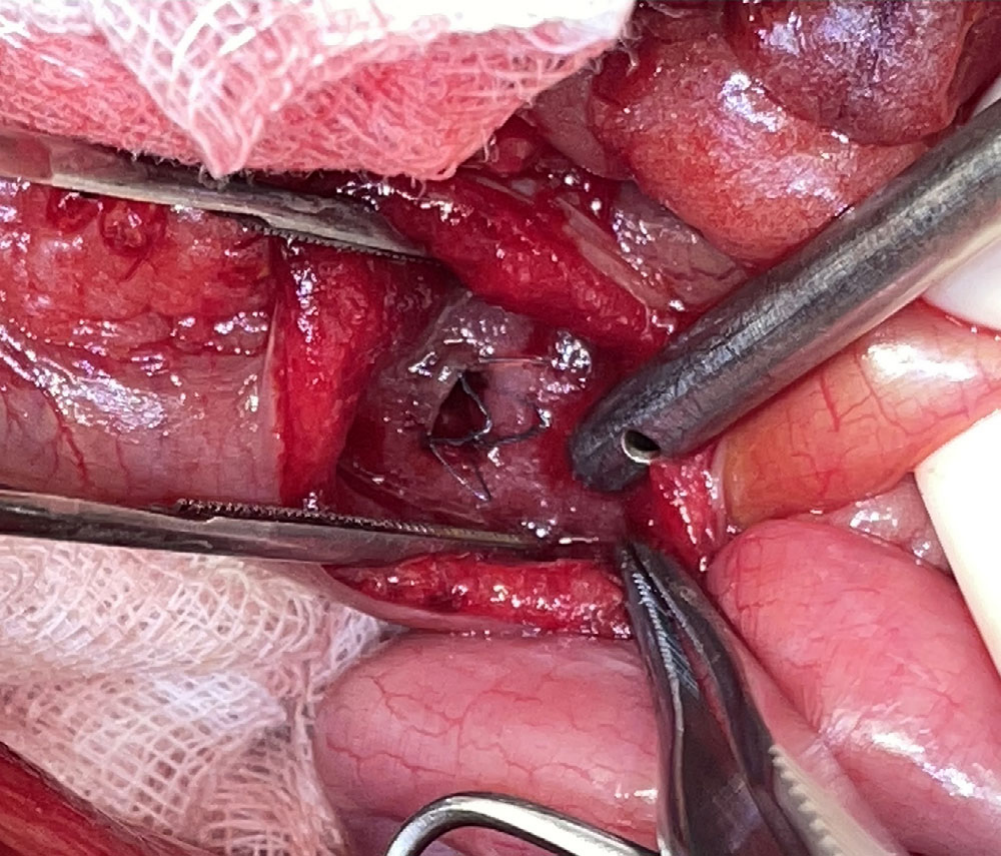
Intraoperative photograph of a self‐expandable metal stent placed within the common bile duct, with the distal end protruding through the major duodenal papilla into the duodenum.

A ventral midline celiotomy was performed. All animals underwent gross examination of the biliary tract to confirm the location and extent of the biliary tract obstruction. A proximal duodenotomy over the anticipated location of the MDP was performed, and SEMS was passed retrogradely through the MDP over a 0.035 mm guidewire. Each SEMS was advanced until its centre reached the site of obstruction and was deployed under fluoroscopic guidance. The most distal portion of the SEMS remained within the duodenum (Figure 2). The patency of CBD was confirmed following SEMS deployment using either a contrast agent or sterile saline before closure of the duodenotomy. All enterotomy sites were closed with 4‐0 polydioxanone suture in a simple interrupted pattern. If indicated, cholecystectomy was performed prior to stent placement. In all patients, the abdomen was lavaged with sterile saline and routinely closed.

## 3 Results

### 3.1 Signalment and Clinical Presentation

Among the 14 retrospectively searched cases of biliary SEMS placement at our referral clinic, 11 dogs and 2 cats satisfied the inclusion criteria. The animals were followed for up to 2 years postoperatively.

Four Poodles, two Maltese, one Bichon Frise, one Coton de Tulear, one Cocker Spaniel, one Shih Tzu, one mixed‐breed dog and one Scottish Straight and one Himalayan cats were included in the study. No breed predilection was found among the animals. The average age was 9.3 years (range, 6–15). A total of 11 were castrated males, and two were spayed females. The average body weight was 5.7 kg (range, 2.3–12.1).

With the exception of one dog who had no symptoms, all animals demonstrated nonspecific clinical signs of EHBO, including inappetence, vomiting and lethargy. Six dogs showed icterus upon physical examination. All 13 patients were diagnosed with EHBO with different underlying causes. Two of the patients had biliary tract obstruction caused by duodenal adenocarcinoma, while another two were diagnosed with cholelithiasis in the CBD. The remaining nine patients were diagnosed with cholangiohepatitis accompanied by biliary sludge and mucus accumulation in the CBD and strictures of the CBD and the MDP. Among these, one patient was diagnosed with hepatocellular carcinoma. All patients had received medical treatment before undergoing SEMS intervention. Cholecystectomy had been performed in previous surgeries before SEMS placement in two animals and concurrently with SEMS placement in six animals. The remaining five patients underwent SEMS placement alone, without cholecystectomy.

### 3.2 Preoperative Diagnostic Tests

Preoperative ultrasound was used to evaluate the presence of concurrent pancreatitis and hepatobiliary diseases. The extent of EHBO was evaluated based on the dilatation and the level of obstruction within the CBD (Table 1).

**TABLE 1:** Signalment and preoperative ultrasonographic evaluation of EHBO.

Case	1	2	3	4	5
**Signalment**	Maltese, MC, 8 years	Bichon Frise, MC, 8 years	Poodle, MC, 6 years	Pompitz, MC, 9 years	Scottish Straight, MC, 8 years
**Pancreatitis**	Y	N	Y	Y	Y
**Choledocholiths**	Y	Y	N	N	N
**CBD diameter**	10 mm	stricture	3.3 mm	8 mm	stricture
**Level of obstruction**	MDP	CBD	MDP	MDP	CBD

Abbreviations: CBD, Common bile duct; EHBO, Extrahepatic biliary obstruction; MC, Male castrated; MDP, major duodenal papilla.

Preoperative serum chemistry revealed marked elevation of liver enzymes (e.g., AST, ALP and GGT) and hyperbilirubinemia in all animals (Table 2).

**TABLE 2:** Pre‐ and postoperative serum liver enzyme and albumin levels.

	*n*	T.bil (mg/dL)	ALT (U/L)	ALP (U/L)	GGT (U/L)	Albumin (g/dL)
**RR**		0.1–0.4	17–78	47–254	5–14	2.6–4.0
**Preoperative**	13	7.36 (0.1–18.8)	1109 (171–3000)	5293 (295–10500)	140 (13–305)	2.83 (2.1–3.8)
**24–48 h**	12	4.76 (0.5–12.9)	696 (243–1050)	5183 (63–11469)	87.8 (19–155)	2.29 (1.4–3)
**1–2 weeks**	10	1.78 (0.2–5.9)	332 (34–1000)	3143 (63–10500)		
**3–4 weeks**	5	0.74 (0.4–1.2)	199 (38–394)	2597 (100–9424)		
**8–16 weeks**	4	0.44 (0.2–0.8)	284 (30–1151)	2153 (97–10402)		
**17–52 weeks**	5	0.45 (0.4–0.5)	307 (60–832)	2745 (78–8171)		

Abbreviations: Post, postoperative; Pre, preoperative; RR, reference range; T. bil, total bilirubin.

### 3.3 Stenting Procedure and Results

**FIGURE 3:**
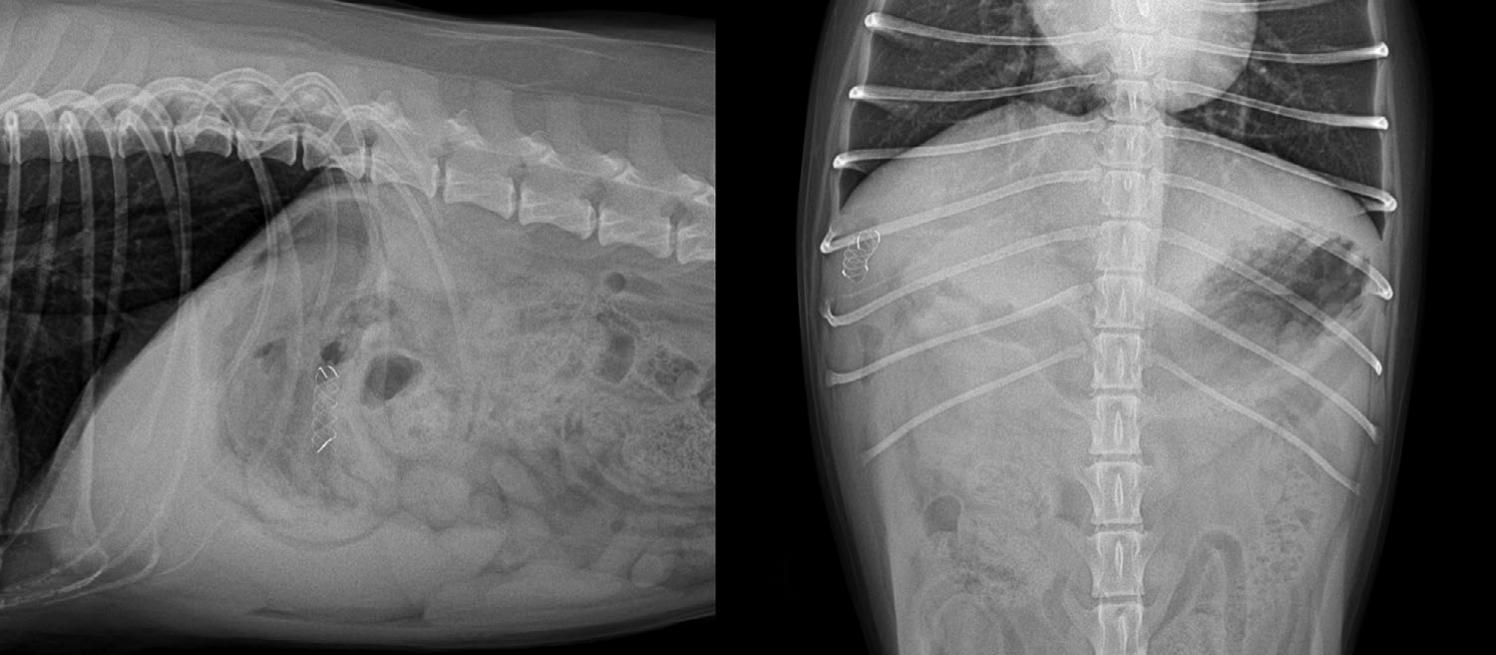
Postoperative abdominal (A) lateral and (B) ventrodorsal radiographs of a dog with a self‐expandable metal stent applied within the common bile duct.

In one dog, an 8 mm × 40 mm (diameter × length), double bare human‐SEMS (EGIS biliary bare stent, S&G Biotech., Seoul, Korea) was used, and the rest of the animals received a 6 mm × 20 mm, single bare animal‐SEMS (Biliary Stent for Animal, S&G Biotech., Seoul, Korea; Figure 3).

Postoperative ultrasound confirmed the resolution of biliary obstruction and restoration of bile flow in all animals immediately after SEMS placement. A decrease in the total bilirubin level was also observed in all patients on the next postoperative day (Table 2).

The animals included in the study were followed for up to 2 years postoperatively. No complications associated with SEMS placement, such as stent migration, bile duct rupture or re‐obstruction, were found in all animals treated with the animal‐SEMS during the follow up periods.

## 4 Discussion

Biliary stenting is indicated in EHBO cases where the patency of CBD is not achieved after medical treatment or permanent rerouting of the biliary tract via cholecystoenterostomy is not an option, especially where bile duct patency is not sustained after cholecystectomy due to remaining biliary debris and stricture caused during CBD lavage (Griffin et al. 2021). In the current study, EHBO originated from both benign and malignant aetiologies. Benign mucocele or sludge found within the GB and extrahepatic biliary tract, especially the CBD, was found in 11 cases in the current study. This led to primarily performing cholecystectomy prior to SEMS placement in eight animals, as cholecystectomy is the established treatment of choice in such cases. In the eight cases where SEMS was added as an auxiliary option, candidates were selected based on the degree of CBD dilatation, extent of sludge/mucus build‐up within the CBD and potential for reobstruction of CBD after cholecystectomy and CBD lavage. Previous studies have described high occlusion and migration rates following red rubber and polyethylene (Boulay et al. 2010). The inconsistent fixation of red rubber stents to the duodenal mucosa and their early dislodgement often lead to re‐obstruction before the resolution of EHBO is achieved. A recent publication by Bergen et al. (2023) described the use of uncovered balloon‐expandable metallic biliary stents (BEMBS) and suggested promising outcomes of using metallic stents, including longer patency and lower occlusion rates. Also, in humans, a mean patency time of 190 days (range 3–586 days) for SEMS was reported (Bergen et al. 2023). Based on these previous studies, the authors hypothesized that the interventional procedure using a novel biliary SEMS is a reliable treatment option for small animals with EHBO.

The animals included in the present study were primarily middle‐aged castrated males weighing less than 9 kg. Most of the animals presented with non‐specific clinical signs of EHBO, such as inappetence, lethargy and vomiting. Despite substantial increases in preoperative total bilirubin and liver enzymes in all animals, icterus on physical examination was found in six (46.1%) animals. Ultrasonography confirmed the diagnosis of EHBO and concurrent pancreatic and hepatobiliary diseases, including pancreatitis, cholangitis, gallbladder mucocele, cholelithiasis, hepatic and duodenal neoplasia.

SEMS placement was successful in all 13 animals in the short term, as evidenced by the postoperative ultrasound imaging of the restoration of bile passage through the CBD, decreases in liver enzyme levels and the alleviation of clinical signs in the immediate postoperative period (Griffin et al. 2021; Silvis et al. 1994). Also, all animals treated with the human‐ or animal‐ biliary SEMS did not experience any of the adverse events associated with red rubber stenting and cholecystoenterostomy, including stent migration, passage, reobstruction, recurrence of EHBO and bile leakage in the perioperative period (Griffin et al. 2021).

The selection of SEMS must be meticulous based on the patient's body weight and the location and extent of the obstruction within the CBD (Lee et al. 2016; Silvis et al. 1994; Lee et al. 2009; Cardella et al. 1995; Lee et al. 2014; Yasumori et al. 1993). In all but one case included in the present study, 6‐mm (diameter) × 20 mm (length) × 4 mm (cell size) animal SEMS was used, which had been developed to improve the duration and efficacy of conventional red rubber and polyethylene stents. This novel device was modified from a commercially available human SEMS in terms of diameter, length and cell size to accommodate the species differences (Nam and Kang, 2016). The diameter in the novel SEMS was decreased, evidenced by the decrease in ascending infection cases following the modification of SEMS diameter from the commonly used 8–6 mm. Stent lengths were modified to include the span of CBD and MDP embedded in the duodenal wall, as MDP stricture occurs more frequently in dogs and cats in comparison to human EHBO cases. Cell sizes were increased in the animal SEMS in order to avoid food boli adhering to the stent lumen, as the distal portion of SEMS is placed within the duodenum past the MDP. In addition, because of the proximity of bile and pancreatic ducts within the MDP in dogs and cats, the use of bare stents, in preference to covered stents, was promoted to avoid possible obstruction of the pancreatic duct by the stent (Mayhew et al. 2006; Berent et al. 2015). In the current study, apart from the earliest case that adopted an 8 mm (diameter) × 40 mm (length) double bare SEMS, which had been previously designed for use in humans, all animals employed the novel 6 mm (diameter) × 20 mm (length) single bare SEMS specifically modified for use in small animals. In the current study, the selection of SEMS was deemed appropriate in all cases that used the novel animal SEMS, supported by the absence of postoperative complications reported in human literature such as persistent ascending cholangiohepatitis and stricture formation (Boulay et al. 2010). Mild elevations in the immediate postoperative liver and acute inflammatory protein levels were found in the case that used the 8 mm human‐SEMS. This was assumed to be associated with transient ascending infections through the larger‐diameter SEMS. Similar complications were not found in the remaining animals that used animal‐SEMS with smaller (6 mm) diameters. All animals included in the study were prescribed liver supplements along with antibiotics up to 16 weeks postoperatively. However, in postoperative Weeks 17–52, a general increase in the liver enzymes were observed. This turnover of liver values during the long term follow‐up was assumed to be partially associated with the progression of chronic and geriatric hepatopathy following the termination of liver supplements.

In the present study, the percentage of dogs and cats with EHBO that required biliary stenting is not known. In addition, no conclusive evidence on the long‐term complications of biliary SEMS placement in animals was found in this study, potentially because of the paucity of long‐term follow‐up after biliary SEMS placement. In addition, owing to the small sample size, statistical analysis of the results could not be performed.

To the best of our knowledge, this is the first case series in the veterinary literature to discuss the use of biliary SEMS as part of the management of EHBO in dogs and cats. In animals that present with EHBO that is amenable to surgical treatment, biliary SEMS placement should be considered as a permanent form of biliary obstruction relief. The procedure is simple, but SEMS remains permanently within the CBD without significant complications, providing palliation of clinical signs and metabolic derangements. A limitation of this study is the small sample size. Additional studies with larger numbers of dogs and cats undergoing biliary SEMS placement are required to obtain more information and to compare the benefits and disadvantages of this procedure with other surgical forms of EHBO treatment. In conclusion, this animal‐specific SEMS may offer one of the alternative methods for managing biliary tract occlusion in the treatment of EHBO.

## Author Contributions

**Suh Hyun Chai**: conceptualization, data curation, investigation, methodology, visualization, writing – original draft, writing – review and editing. **Kyuseok Choi**: conceptualization, investigation, methodology, writing – review and editing. **Sungin Lee**: conceptualization, data curation, writing – original draft, writing – review and editing. **Yongsun Kim**: conceptualization, data curation, investigation, methodology, visualization, writing – original draft, writing – review and editing.

## Ethics Statement

The authors confirm that the ethical policies of the journal, as noted on the journal's author guidelines page, have been adhered to. No ethical approval was required as this is a retrospective article with no original research data.

## Conflicts of Interest

The authors declare no conflicts of interest.

## Peer Review

The peer review history for this article is available at https://publons.com/publon/10.1002/vms3.70548.

## Data Availability

Data openly available in a public repository that issues datasets with DOIs.
